# Supplementary material for: Single-shot ultrafast terahertz photography
Source: Nat Commun. 2023 Mar 27;14:1704. doi: 10.1038/s41467-023-37285-3 (PMC10042990; doi:10.1038/s41467-023-37285-3)
Supplement: Supplementary file 1 — Supplementary Information [file 41467_2023_37285_MOESM1_ESM.pdf]

Supplementary Information

Single-shot ultrafast terahertz photography

***Junliang Dong*[1,†,*]*, Pei You*[1,†]*, Alessandro Tomasino*[1,†]*, Aycan Yurtsever*[1]*, and Roberto Morandotti*[1,*]**

[1.] *Institut national de la recherche scientifique, Centre Énergie Matériaux Télécommunications, Varennes, QC J3X 1P7, Canada.*

[†] *These authors contributed equally.*

[*] *Corresponding authors: J.D.: Junliang.Dong@inrs.ca and R.M.: Roberto.Morandotti@inrs.ca*

Supplementary Note 1: Spatial resolution and post-processing procedure

Here, we discuss the spatial resolution of our single-shot ultrafast photography system and provide more detailed information about the post-processing procedure for the reconstruction of individual frames.

In order to assess the system's spatial resolution, we imaged a dynamic scene, which was a THz pulse propagating through a Teflon sheet (thickness: 0.8 mm) with a pinhole (diameter: 2 mm) in the center, as depicted in Supplementary Fig. 1. Based on the imaging system illustrated in Fig. 2 of the main manuscript, this dynamic scene carried by the THz waves was relayed onto the ZnTe crystal by means of the lenses TPX2 (focal length: 100 mm) and TPX3 (focal length: 65 mm). The electric-optic sampling (EOS) technique for THz detection was operated in a co-propagating configuration. Before imaging, the THz waveforms with and without the sample were recorded and are plotted in Supplementary Fig. 1b. By referring to the temporal evolution of the THz waveforms, we set the arrival time of the multiplexed probe beam to 4.60 ps with an inter-frame time interval of 0.60 ps to capture representative frames of the THz wave propagating through the pinhole.

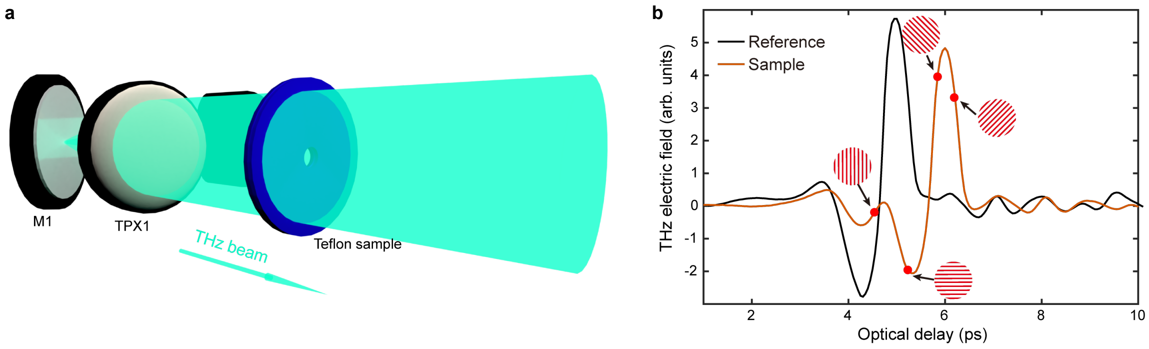

Supplementary Figure 1. a Schematic representation of the experimental setup for the collimated THz beam used to capture the dynamic scene. It consists of a THz pulse passing through a Teflon sheet (thickness: 0.8 mm) with a pinhole (diameter: 2 mm) in the center. **b** THz waveforms acquired via the EOS technique with and without the Teflon sample.

The multiplexed image captured by the CCD camera is shown in Supplementary Fig. 2a. Despite being overlapped in the spatial domain, these frames are well separated in the Fourier domain, as shown in Supplementary Fig. 2b. As such, each of them can be extracted and computationally recovered. Here, we take the recovery of the fourth frame (the frame taken at 6.40 ps) as an example to demonstrate the post-processing procedure. After applying the 2D Fourier transform, one image copy of the fourth frame in the Fourier domain was selected using a spatial-frequency band-pass filter, as shown in Supplementary Fig. 2c. Specifically, a 2D band-pass with a rectangular window was used in our study. After applying such a band-pass filter, the spatial-frequency content of the fourth frame was isolated and extracted, in turn removing all the other

image copies, as shown in Supplementary Fig. 2d. Upon the knowledge of the modulation frequency, the isolated frequency content was then digitally transferred to the center (origin) of the Fourier domain, as shown in Supplementary Fig. 2e, in turn transforming the sinusoidal modulation into a uniform illumination in the real space. In the final step, by applying the inverse Fourier transform on this filtered and rearranged spectrum, the fourth frame that was encoded at the shifted region in the Fourier domain was recovered, as shown in Supplementary Fig. 2f.

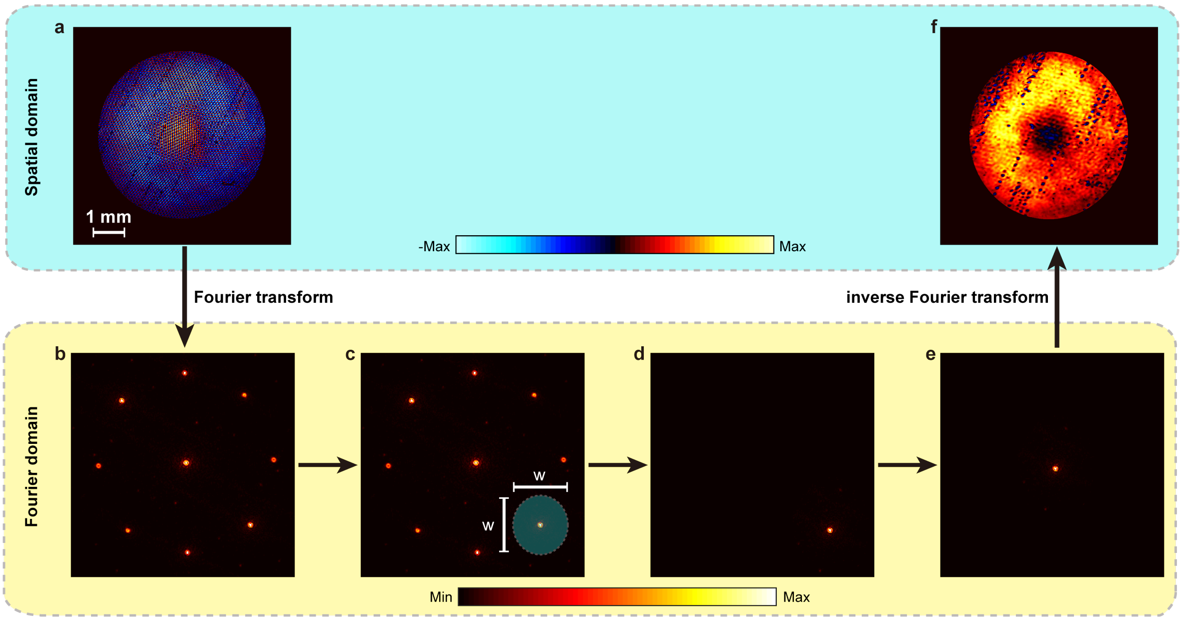

Supplementary Figure 2. Post-processing procedure. **a** Raw multiplexed image captured by the CCD. **b** 2D Fourier transform of the image in **a**. **c** A 2D band-pass filter with a width of w is used to select an individual frame. The width w chosen here is 140 pixels in the Fourier domain. **d** The band-pass filter is multiplied by the Fourier transform of the raw image, removing the other image copies. **e** The filtered frequency content is shifted to the center of the Fourier domain, which transforms the sinusoidal modulation into a uniform illumination. **f** The original frame is recovered by applying the inverse Fourier transform of the filtered and rearranged spectrum in **e**.

It is important to note that the width of the filtering window (in pixels) in the Fourier domain may influence the spatial resolution of the recovered frame. In principle, the larger the window width, the more the frequency content is preserved, because any spatial-frequency information outside the window is discarded. Supplementary Figs. 3a-3f display the fourth frames that were extracted and recovered using different widths of the band-pass filter. Indeed, it is clear that as the width increases, more features, such as the small voids in the ZnTe crystal, can be identified in the recovered frame. In addition, the width of the filter should also be small enough to avoid cross-talk or interference with the image copies of adjacent frames. As shown in Supplementary Fig. 3f, when the width (in pixels) is equal to 170, interference among different frames is observed. The spatial resolution can be characterized by investigating the edge-response of the pinhole in the recovered THz frame. The width of the edge-response was calculated by measuring the distance between the points at 10% and 90% of its peak value. Supplementary Fig. 3g displays the THz

electric field distribution across the white dashed line plotted in Supplementary Fig. 3a. It is found out that the width of the band-pass filter does not affect the spatial resolution estimation. This is because the feature of the pinhole was captured by recording the THz image in a far-field configuration, in which the spatial resolution follows the diffraction limits criterion, i.e., it mainly depends on the central frequency of the employed THz source. By operating the EOS technique with an optical probe beam at 800 nm, the spatial resolution achieved is 0.56 mm, consistently with the central frequency of our THz source (~0.5 THz), as shown in Supplementary Fig. 3h. Such a spatial resolution can be still preserved when the width of the band-pass filter is equal to 20. However, the width of the filter has an impact on the subtle features captured by the optical probe. This is the reason why small voids in the ZnTe crystal can be identified as we increased the filter width.

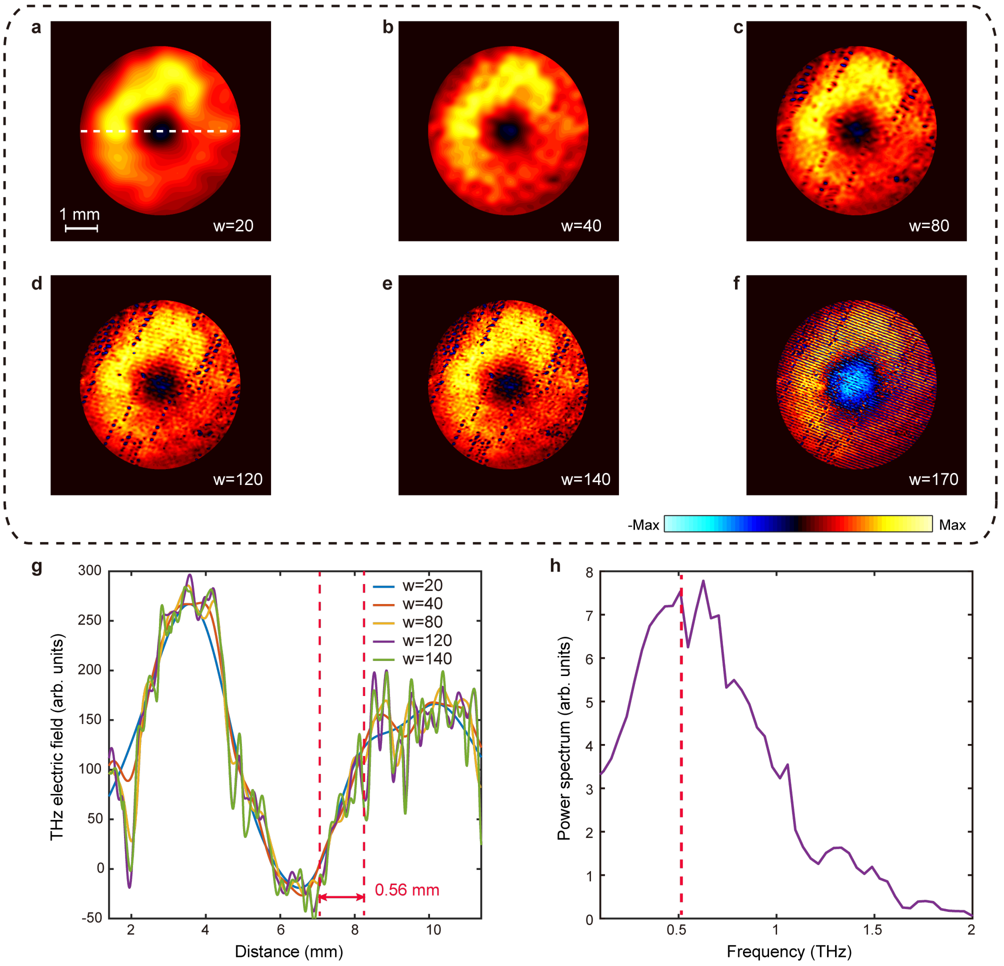

Supplementary Figure 3. Spatial resolution of the recovered frame. **a-f** Recovered frames when the width (in pixels) of the band-pass filter is equal to 20, 40, 80, 120, 140, and 170, respectively. **g** THz electric fields distribution across the white dashed line in **a**, obtained using different widths of the band-pass filter. **h** Spectrum of the THz pulse generated from our $LiNbO_3$ source.

Following the post-processing procedure, the four frames were extracted and individually reconstructed when the width of the band-pass filter was equal to 140. The recovered frames, corresponding to the 2D distribution of the THz electric field at four specific times, are shown in Supplementary Fig. 4a. As expected, the central part of the THz beam that directly travelled through the pinhole arrived first. This is because THz waves travel faster in air than in the Teflon sheet. By changing the arrival time of the multiplexed probe beam, the dynamic scene at other time positions can also be obtained. Supplementary Figures 4b and 4c display another two sets of frames that were captured when the probe beam was temporally shifted by ±50 fs relative to the case in Supplementary Fig. 1b. These time-lapse frames clearly reveal the spatial and temporal evolution of a bipolar THz pulse passing through the sample, based on its distinct propagation speeds in different materials.

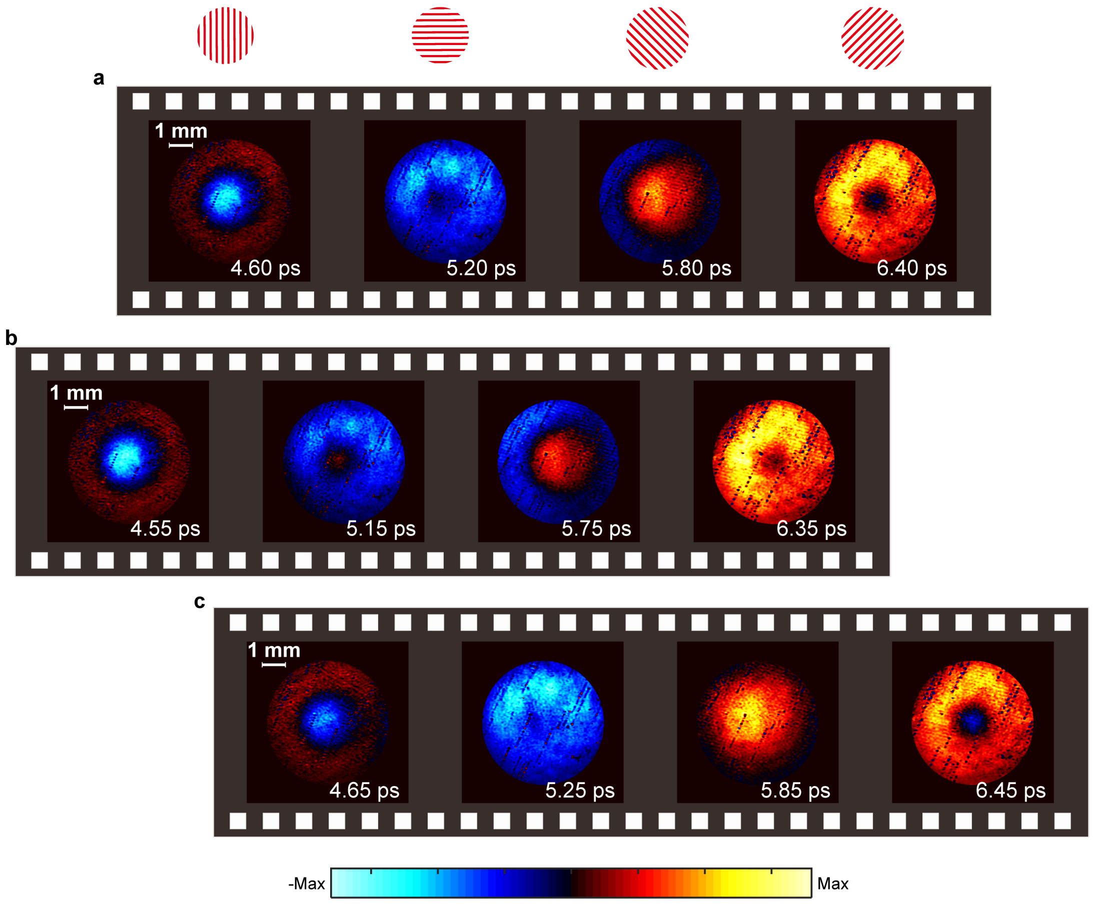

Supplementary Figure 4. a Recovered frames from the multiplexed image in Supplementary Fig. 2a. **b** Recovered frames that were captured when the probe beam arrived 50 fs earlier relative to the case in Supplementary Fig. 1b. **c** Recovered frames that were captured when the probe beam arrived 50 fs later relative to the case in Supplementary Fig. 1b. The image contrast of the frames is given by the amplitude of the THz electric field.

Supplementary Note 2: Maximum number of frames

Here we discuss the theoretical limit for the number of frames that can be multiplexed in our imaging system. The principle at the core of spatial-frequency multiplexing is to shift the band-limited frequency content of the image to unexploited regions in Fourier space, by applying distinct modulation patterns onto the object. In order to estimate the maximum number of frames than can be multiplexed, we need to consider two important parameters[1]: 1) the overall area of the unexploited region in Fourier space $A_{unexploited}$ which is available for multiplexing, and 2) the minimum area that can be in Fourier space for the recovery of each frame, A_{filter}. Fixing this lower limit avoids overlapping between adjacent frames, while at the same time guaranteeing a satisfactory spatial resolution of the recovered image.

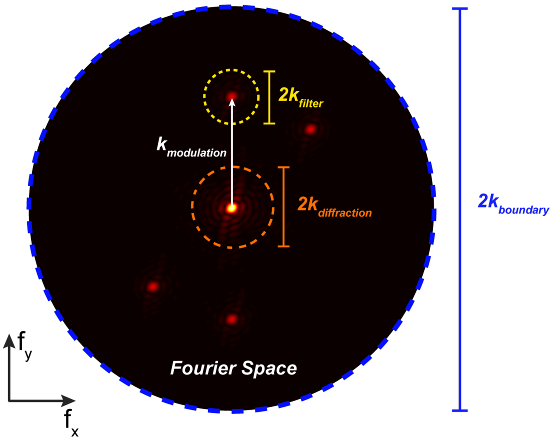

Supplementary Figure 5. Schematic of the Fourier domain used to estimate the maximum frame numbers.

Fourier space is considered as a circle with a radius of $k_{boundry}$, which is determined by the pixel size and number of pixels in the camera, see Supplementary Fig. 5. The frequency content of the original image is band-limited and only occupies the central region with a radius of $k_{diffraction}$ in Fourier space. $k_{diffraction}$ is determined by the spatial resolution of the image subject to the diffraction limit. Therefore, the unexploited region available for multiplexing is typically an annulus that can be calculated as

$$A_{unexploited} = \pi k_{boundary}^2 - \pi k_{diffraction}^2 . \qquad (1)$$

By illuminating the object using sinusoidal modulation with a spatial-frequency $k_{modulation}$, a pair of image copies of the original image occur at $\pm\, k_{modulation}$ in Fourier space. To recover the frame, one image copy is isolated using a spatial-frequency band-pass filter with a radius of k_{filter}, which determines the spatial resolution of the recovered frame. The area in Fourier space that is extracted for this frame recovery is calculated as:

$$A_{filter} = \pi k_{filter}^2 . \qquad (2)$$

The upper limit on the number of multiplexed frames N_{max} can be estimated using the ratio between the area of the annulus and the area of the filter:

$$N_{max} = \left\lfloor \frac{A_{unexploited}}{2A_{filter}} \right\rfloor = \left\lfloor \frac{k_{boundary}^2 - k_{diffraction}^2}{2k_{filter}^2} \right\rfloor . \qquad (3)$$

The factor of 2 accounts for the image copies that occur in Fourier space, which appear symmetrically with respect to the origin. It is important to note that the maximum number of frames estimated by Eq. 3 is based on the assumption that all possible modulation patterns with different periods and orientations can be employed, which is actually an extreme case. In our study, spatial-frequency multiplexing was achieved by simply rotating the orientation while keeping the modulation period constant. In this case, the maximum number of frames is calculated as:

$$N_{max} = \left\lfloor \frac{\left(k_{modulation} + k_{filter}\right)^2 - \left(k_{modulation} - k_{filter}\right)^2}{2k_{filter}^2} \right\rfloor ,$$

with $\left(k_{diffraction} + k_{filter}\right) \leq k_{modulation} \leq \left(k_{boundary} - k_{filter}\right)$. (4)

It is obvious that there is a trade-off between N_{max} and k_{filter}, which indicates that we have to sacrifice some image quality if more frames are required. In our experiments, we have Fourier space with a radius of 521 pixels while the length of $k_{modulation}$ is 102 pixels, based on the modulation patterns we applied. As previously discussed, the spatial resolution of the THz image, which is subject to the diffraction limit, can still be preserved when k_{filter} is equal to 10. Therefore, based on Eq. 4, the maximum number of frames that can be multiplexed is 20. If k_{filter} is increased in order to see subtle details in the frames (e.g., $k_{filter} = 20$), then the maximum number of frames decreases to 10. For the extreme case in Eq. 3, where all possible modulation patterns with different periods and orientations can be implemented, more than 1000 frames can be multiplexed when k_{filter} is equal to 10.

Supplementary Note 3: Numerical simulations of photo-excited carrier dynamics in bulk silicon

Here, we analytically investigate the transient response of photo-excited carriers in bulk silicon (Si), upon an oblique illumination of an ultrafast optical pulsed beam. We corroborate the experimental results demonstrated in Fig. 4 of the main manuscript by comparing them with the simulated spatial and temporal dynamics of the transient captured in an interval of several picoseconds after photoexcitation.

Undoped, high-resistivity Si behaves as a dielectric material at THz frequencies. It has very few dark carriers and shows a dispersion-less refractive index of 3.42 from 0.5 THz to 4.5 THz with minimal absorption[2]. However, if the number of carriers is increased via photo-excitation, Si can

behave as a classical Drude conductor[3]. This allows for the modulation of a THz beam via the optical excitation of Si. In our study, THz transmission through a photo-excited Si wafer is simulated by considering a normally incident THz beam travelling through a multilayered structure, as depicted in Supplementary Fig. 6a. The Si wafer, surrounded by air (refractive index n_0), has a thickness of h and complex refractive index $\tilde{n}(x,y,z)$ at the considered THz frequency. The complex refractive index $\tilde{n}(x,y,z)$ can be obtained by calculating the carrier concentration profile N generated by the photoexcitation and then applying the Drude model, which relates the carrier concentration to a frequency-dependent complex dielectric permittivity[4,5].

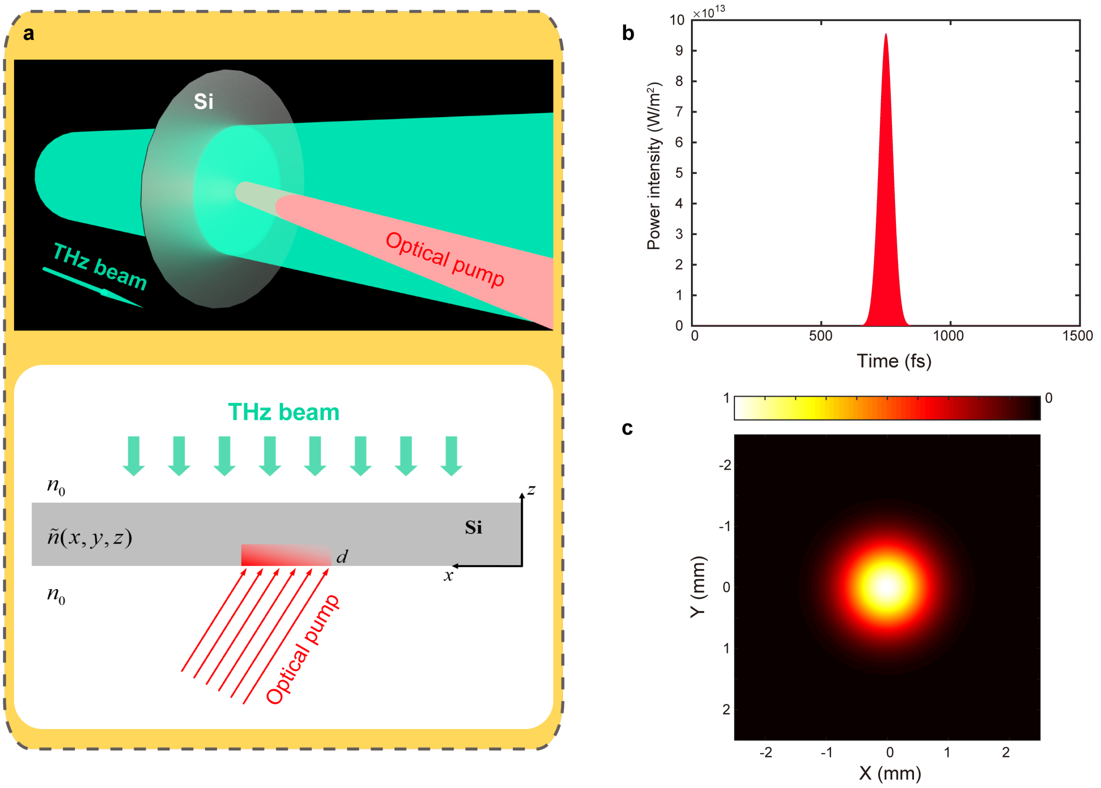

Supplementary Figure 6. a Schematic diagram for the simulated geometry. **b** Temporal profile of the optical pump beam. **c** Normalized spatial profile (Gaussian distribution) of the optical pump beam.

The time representation of the electric field $E(t)$ of a femtosecond optical pulse is given by:

$$E(t) = E_0 e^{-a_0 t^2} e^{-i\omega_0 t}, \tag{5}$$

where ω_0 is the central angular frequency and a_0 is the time constant. The intensity $I(t)$ can then be derived as:

$$I(t) = \frac{1}{2}\varepsilon_0 c \left|E(t)\right|^2 = \frac{1}{2}\varepsilon_0 c E_0^2 e^{-2a_0 t^2}, \tag{6}$$

where ε_0 is the dielectric constant in vacuum and c is the speed of light. In our study, we consider an 800-nm ultrafast laser pulse with a duration (FWHM) of 150 fs. The beam is assumed Gaussian with a 1/e^2 diameter of 2 mm. The temporal and spatial intensity distributions of such an optical pulse are shown in Supplementary Figs. 6b and 6c, respectively. Such a beam illuminates the center of a Si wafer featuring a thickness h = 500 μm, impinging with an oblique incidence of 30°. The average power of the optical beam is 50 mW.

When the optical pulse starts to illuminate the Si wafer, the absorption of photons creates electron-hole pairs in the wafer with generation rate g and carrier lifetime τ. The carrier generation rate g decays exponentially along the z-direction and can be expressed as[6]:

$$g\left(x,y,z,t\right)=\alpha I(x,y,t)\left(1-R\right)e^{-\alpha z}\big/\hbar\omega_0\,, \tag{7}$$

where α is the absorption coefficient of the Si wafer at the illumination frequency ω_0, while $\hbar\omega_0$ is the photon energy. R = 0.275 is the reflectivity at the air/Si interface, while the penetration depth d in Si is about 12 μm at 800 nm[7] (the absorption coefficient α is calculated using $\alpha = 1/d$). Upon optical illumination, the photo-excited carrier concentration profile $N(x,y,z,t)$ in the Si wafer can be calculated by using the relation[6]:

$$\frac{\partial N}{\partial t}=D_{eff}\nabla^2 N-\frac{N}{\tau}+g\,, \tag{8}$$

where D_{eff} is the effective carrier diffusion coefficient. The carrier lifetime τ in Si is 25 μs[8]. In our study, we focus on the transient response of the carriers within several picoseconds after photo-excitation, therefore, we can neglect carrier diffusion and recombination effects and only take the carrier generation rate g into account. As a result, the transient carrier concentration profile N at a given time t is expressed as[6]:

$$N\left(x,y,z,t\right)=\int_0^t \alpha I(x,y,\eta)\left(1-R\right)e^{-\alpha z}\big/\hbar\omega_0 d\eta\,. \tag{9}$$

The achieved carrier concentration N is then exploited to calculate the corresponding frequency-dependent complex dielectric constant using the Drude model[5]:

$$\varepsilon\left(\omega\right)=\varepsilon_\infty-\frac{Ne^2}{\varepsilon_0 m\omega^2}=\varepsilon_\infty-\frac{\omega_p^2}{\omega^2}\,, \tag{10}$$

where $\omega_p=\sqrt{Ne^2/m\varepsilon_0}$ is known as the plasma angular frequency. Furthermore, the complex refractive index $\tilde{n}(x,y,z)$ of the photo-excited Si wafer at a THz frequency ω can be calculated using $\tilde{n}=\sqrt{\varepsilon}$. Based on the equations provided above, the transmission of a normally incident THz beam through the photo-excited Si wafer can be obtained by using the Fresnel equation[5].

In our simulations, we consider a geometrical domain of 6 mm by 6 mm, assuming a (uniform) THz beam size and 0.4 THz as the frequency of interest. The grid unit in space is 5 μm by 5 μm with a time step of 1 fs, and the total simulation time length is 10 ps. The achieved results are shown in Supplementary Fig. 7. The THz transmission curve depicts the energy of the transmitted

THz beam as the optical pump arrives at the Si wafer. When the optical pump strikes on the wafer, we start to observe a decrease in THz transmission, as photoexcitation leads to the generation of carriers, thus changing the conductivity. The decrease lasts for ~7 ps until the carriers are fully excited within the illumination area. The total decrease time is mainly determined by the geometry and the incident angle of the optical beam. Typical THz images in transmission at different time instants are also shown in the insets of Supplementary Fig. 7. Such images reflect the evolution of photoexcitation as a function of the local arrival time of the optical pump beam, impinging with an oblique incidence. It is noteworthy to emphasize that a slight variation in the size, shape, and the incident angle of the optical pump beam will influence both the temporal and spatial dynamics of the photo-excited carriers. This also explains the slight deviations between the simulations and the experimental results presented in the main manuscript.

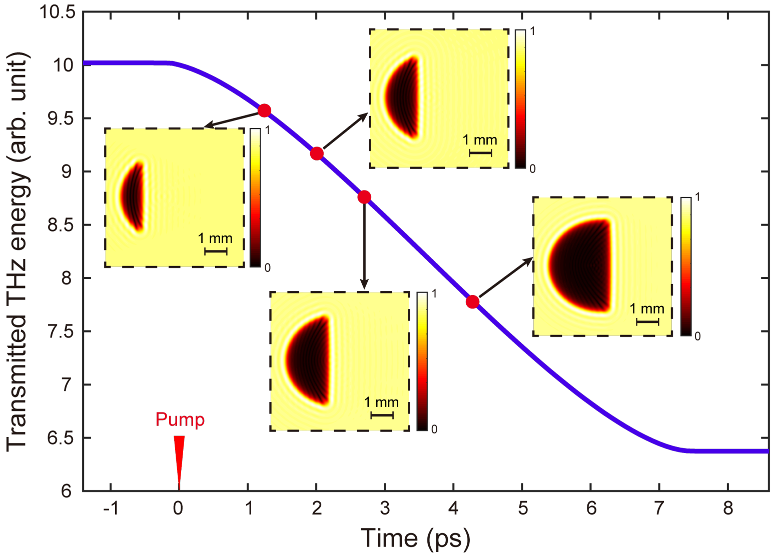

Supplementary Figure 7. Simulated THz transmission curve when the optical pump illuminates the Si wafer with an oblique incidence. The insets show the simulated (and normalized) THz images in transmission at 1.25 ps, 2.00 ps, 2.75 ps, and 4.10 ps after photoexcitation, respectively, as observed with our ultrafast THz photography system.

Supplementary References:

1. Gragston, M., Smith, C., Kartashov, D., Shneider, M. N. & Zhang, Z. Single-shot nanosecond-resolution multiframe passive imaging by multiplexed structured image capture. *Opt. Express* **26**, 28441 (2018).
2. Dai, J., Zhang, J., Zhang, W. & Grischkowsky, D. Terahertz time-domain spectroscopy characterization of the far-infrared absorption and index of refraction of high-resistivity, float-zone silicon. *J. Opt. Soc. Am. B* **21**, 1379 (2004).
3. Jeon, T.-I. & Grischkowsky, D. Nature of Conduction in Doped Silicon. *Phys. Rev. Lett.* **78**, 1106–1109 (1997).
4. Steinbusch, T. P., Tyagi, H. K., Schaafsma, M. C., Georgiou, G. & Gómez Rivas, J. Active terahertz beam steering by photo-generated graded index gratings in thin semiconductor films. *Opt. Express* **22**, 26559 (2014).
5. Ulbricht, R., Hendry, E., Shan, J., Heinz, T. F. & Bonn, M. Carrier dynamics in semiconductors studied with time-resolved terahertz spectroscopy. *Rev. Mod. Phys.* **83**, 543–586 (2011).
6. Kannegulla, A., Shams, M. I. Bin, Liu, L. & Cheng, L.-J. Photo-induced spatial modulation of THz waves: opportunities and limitations. *Opt. Express* **23**, 32098 (2015).
7. Green, M. A. Self-consistent optical parameters of intrinsic silicon at 300K including temperature coefficients. *Sol. Energy Mater. Sol. Cells* **92**, 1305–1310 (2008).
8. Gaubas, E. & Vanhellemont, J. Comparative Study of Carrier Lifetime Dependence on Dopant Concentration in Silicon and Germanium. *J. Electrochem. Soc.* **154**, H231 (2007).
